# CRAC (Clinical Relevance of Alterations in Cancer): a Knowledge Base for the Selection of Molecularly Matched Therapy for Solid Tumors

**DOI:** 10.17691/stm2022.14.6.02

**Published:** 2022-11-28

**Authors:** A.A. Lebedeva, A.I. Kavun, E.M. Veselovsky, V.A. Mileyko, M.V. Ivanov

**Affiliations:** Head of Data Interpretation Group; Oncoatlas LLC, 31 Malaya Nikitskaya St., Moscow, 121069, Russia;; Senior Data Interpreter; Oncoatlas LLC, 31 Malaya Nikitskaya St., Moscow, 121069, Russia;; Technology Development and Integration Specialist; Oncoatlas LLC, 31 Malaya Nikitskaya St., Moscow, 121069, Russia;; Director; Oncoatlas LLC, 31 Malaya Nikitskaya St., Moscow, 121069, Russia;; Head of the Research and Development Department; Oncoatlas LLC, 31 Malaya Nikitskaya St., Moscow, 121069, Russia; Researcher, School of Living Systems; Moscow Institute of Physics and Technology (National Research University), 9 Institutskiy per., Dolgoprudny, Moscow Region, 141701, Russia

**Keywords:** database, precision oncology, NGS, clinical interpretation, molecularly matched therapy

## Abstract

Multigene testing using NGS (next-generation sequencing) provides a large amount of information and can detect multiple molecular alterations. Subsequent clinical interpretation is a time-consuming process necessary to select a treatment strategy. Existing databases often contain inconsistent information and are not regularly updated. The use of ESCAT levels of evidence requires a deep understanding of the nature of alterations and does not answer the question of which therapy option to select when multiple biomarkers with the same level of evidence are detected. To address these issues, we created the Clinical Relevance of Alterations in Cancer (CRAC) database on the relevance of detected alterations in specific genes, which are often analyzed as part of NGS panels. The team of oncologists and biologists assigned a CRAC score from 1 to 10 to each biomarker (a type of genomic alteration characteristic of specific genes) for 15 malignancies; an average score was entered into the database. CRAC scores are a numerical reflection of the following factors: therapy availability and the prospects of drug treatment with experimental drugs for patients with a particular type of tumor. A total of 134 genes and 15 of the most common tumor types have been selected for CRAC. The biomarker–nosology associations with CRAC scores in the range of 1–3 are the most frequent (n=2719 out of 3495; 77.8%), the least frequent ones (n=52 out of 3495; 1.5%) are with the highest CRAC scores 9 and 10. To estimate the practical effectiveness of the CRAC database, 208 reports on comprehensive molecular profiling were retrospectively analyzed; the applicability of CRAC was compared with the ESCAT level of evidence system. The highest CRAC scores corresponded to the ESCAT maximum levels of evidence: the range of scores 8–10 corresponded to evidence levels I and II. No biomarker within the same level of evidence was represented by the same CRAC score; the largest range of CRAC scores was observed for biomarkers of levels evidence IIIA and IV — from 2 to 10 and from 1 to 9, respectively. The use of CRAC scores allowed to identify additional 95 alterations with CRAC scores of 1–5 in the studied patients.

The developed database is available at: https://crac.oncoatlas.ru/.

## Introduction

With the growing use of molecular profiling, the approach to drug treatment of various types of tumors is actively changing [1]. The results of NGS-based genomic testing showed that the use of molecularly matched therapy significantly improves the overall survival of patients with various solid tumors [2-4]. Considering the lengthy process of drug approval by regulators for certain indications and their introduction into routine clinical practice, targeted drugs become available in different countries through the active initiation of expanded access (commonly referred to as compassionate use) to therapy programs [5, 6]. Such programs provide receiving promising treatment regardless of indications, including cases when relevant genomic alterations are detected in patients [7].

Molecular profiling using NGS is actively entering international clinical practice and becoming more affordable to patients. The European Society for Medical Oncology (ESMO) recommends the use of multigene NGS screening for a number of tumor types, including non-small cell lung cancer, cholangiocarcinoma, prostate cancer, and ovarian cancer [8]. Such testing yields a massive amount of genomic data and often detects many molecular alterations. The subsequent clinical interpretation of the detected alterations should answer the question of what therapeutic strategies should be used to suppress carcinogenesis in tumor cells with the molecular profile in question, and how effective they will be.

**Figure 1.**
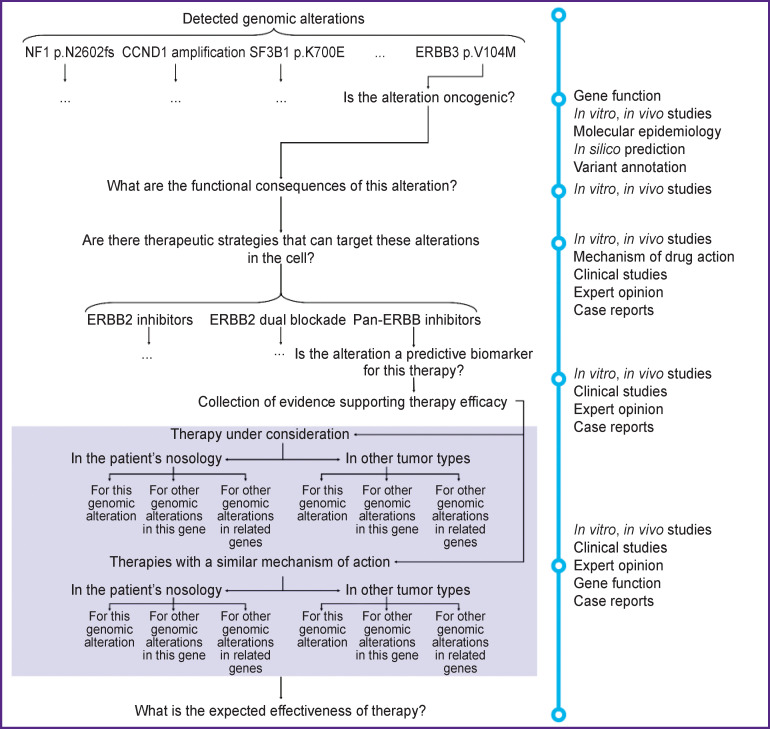
The process of interpreting genomic alterations detected by comprehensive molecular profiling

Clinical interpretation is a complex, multilevel process that requires not only competencies in many branches of knowledge related to oncology, but also an extensive literature search [9] (Figure 1). To make a correct therapeutic decision, it is necessary not only to clinically interpret the results of molecular testing at an appropriate level of quality in accordance with international standards and principles of evidence-based medicine, but also to present them in a concise manner that can be understood by third-party specialists. To systematize all the evidence supporting the effectiveness of a particular therapy, levels of evidence are applied [10, 11], and to simplify the process itself, precision oncology databases are used [10-14].

In addition to the issues associated with the interpretation and determination of the clinical significance of the detected alterations, there are a number of other challenges. Thus, molecular profiling can identify several clinically significant biomarkers, which are promising targets for molecularly matched therapy, a balanced choice between which depends on understanding their association with the effectiveness of a particular therapy, as well as the nature of the alteration. The application of the evidence levels used in practice requires awareness of the nature of the alteration and an extensive and time-consuming literature search. Levels of evidence do not provide a direct answer to the question of which treatment to select in case of detection of several biomarkers of the same level. For example, the existing ESCAT system does not take into consideration specific endpoints when evaluating a response [15]. Knowledge bases on the clinical significance of biomarkers provide information that is close to exhaustive only for biomarkers of evidence levels I and R1, which are used in routine clinical practice. Moreover, such knowledge bases have different contents, and the information contained in them is updated at various intervals [16].

In order to address the above challenges, we created the Clinical Relevance of Alterations in Cancer (CRAC) database on the relevance of detected alterations in specific genes, which are commonly analyzed as part of NGS panels. The database is available at the link: https://crac.oncoatlas.ru/.

## Materials and Methods

### Creation of a database on the clinical significance of alterations

To create a database, 15 common malignancies were selected (breast cancer, ovarian cancer, non-small cell lung cancer, colorectal cancer, prostate cancer, pancreatic cancer, uterine cancer, skin melanoma, cholangiocarcinoma, urothelial cancer, soft tissue sarcomas, salivary gland cancer, CNS tumors, gastric/esophageal cancer, neuroendocrine tumors of the gastrointestinal tract), one of the options of treatment for which is drug therapy.

The genes involved in carcinogenesis of solid tumors (tumor suppressor genes and proto-oncogenes, as well as genes demonstrating suppressor and proto-oncogenic activity) were selected. Alterations that are predictive of the potential efficacy of promising anticancer targeted drugs and/or immune checkpoint inhibitors were preliminarily selected for each of them. Later, the genes were grouped according to their type. Structural alterations leading to the formation of chimeric protein variants (known as translocations or rearrangements), as well as biomarkers associated with resistance to any therapy, were not considered.

Further, a CRAC score from 1 (minimum score) to 10 (maximum score) for each type of clinically significant alterations, depending on the gene and tumor localization was assigned. A CRAC score was a numerical reflection of the following factors: the availability of therapy and the feasibility of drug treatment with experimental drugs for patients with a specific type of tumor.

Thirteen experts participated in the development of the database; on average, 5 people being involved in the evaluation of one biomarker. The team of experts included practicing oncologists and chemotherapists with considerable experience of using the results of molecular profiling to select therapy in their routine practice, as well as biologists involved in the interpretation of the results of molecular profiling. All the specialists involved in filling out the database take part in molecular tumor boards on a regular basis.

When creating the database, we took into account any molecularly matched therapy approved by regulators (FDA, EMA, State Register of Medicines — SRM), as well as any molecularly matched therapy conducted in clinical trials, regardless of whether the presence of a biomarker of interest was an inclusion criterion in this study. For experimental drugs which are not included in any of the above regulators, the maximum stage of ongoing active clinical trials at the time of creating the database was taken into account. The prospects of molecularly matched therapy were determined by experts based on the relevance and prospects of the association between the target and therapy in the absence of published data on the effectiveness of such an approach, and, if available, on the basis of the results presented in the literature, taking into account the level of reliability and consistency between them.

In case the effectiveness of the therapy for any genomic alteration was not sufficiently proven — in accordance with the ESCAT recommendations (either limited or insufficiently studied), that therapy was not entered into the database. The status of alterations (somatic or germline) was not considered. Two groups of experts (molecular biologists and oncologists) independently assigned numerical scores for the predictive role of genomic alterations. The average score was put down into a table, on the basis of which the CRAC database was then created. It is important to note that the occurrence of alterations in a particular type of tumor was not taken into account when developing the database.

### A retrospective analysis of the reports of patients who underwent comprehensive tumor molecular profiling

A retrospective analysis of reports on the results of comprehensive molecular tumor profiling in 208 patients conducted from June 2021 to June 2022 was performed just in order to test the utility of the database, but not for its filling. For comprehensive molecular profiling, the analysis of 150 and more genes via NGS were considered. A FFPE block with the most recent material taken from the primary tumor or metastatic tissue was used for testing.

The detected genomic alterations were analyzed considering the tumor type; the gene that has been altered; the nature and functional consequences of alteration; an analysis of relevant therapeutic strategies and their potential effectiveness. Literature data were used in the interpretation process. Each association of a biomarker with a drug was assigned an appropriate level of evidence according to the ESCAT system [11].

## Results

### Description of the resulting database

A total of 134 genes were selected, including 76 proto-oncogenes and 58 tumor suppressors involved in carcinogenesis. A total of 233 biomarkers were analyzed. The most common alterations were CNV (n=113; 48.5%), any damaging/possibly deleterious genetic variants (n=53; 22.7%), somatic mutations at hotspots (n=22; 9.4%), as well as pathological alterations in certain functionally important domains (n=36; 15.4%). The largest number of alterations (n=6) was marked separately for the *EGFR* gene: deletions in exon 19 and p.L858R; insertions in exon 20; p.S768I, p.L861Q, and p.G719X variants; any somatic variants in the tyrosine kinase domain; any somatic variants in any functionally important domain; copy number variation. The average number of genomic alterations for each gene was 2. For genes showing suppressor activity, damaging genetic variants, as well as copy number variants, were most commonly used.

The median of the maximum CRAC score independent of localization was 7.5 (copy number variants of the *ERBB2* gene), the median of the lowest score was 1 (any damaging/probably damaging genetic variants, for example, for *TP53*).

**Figure 2.**
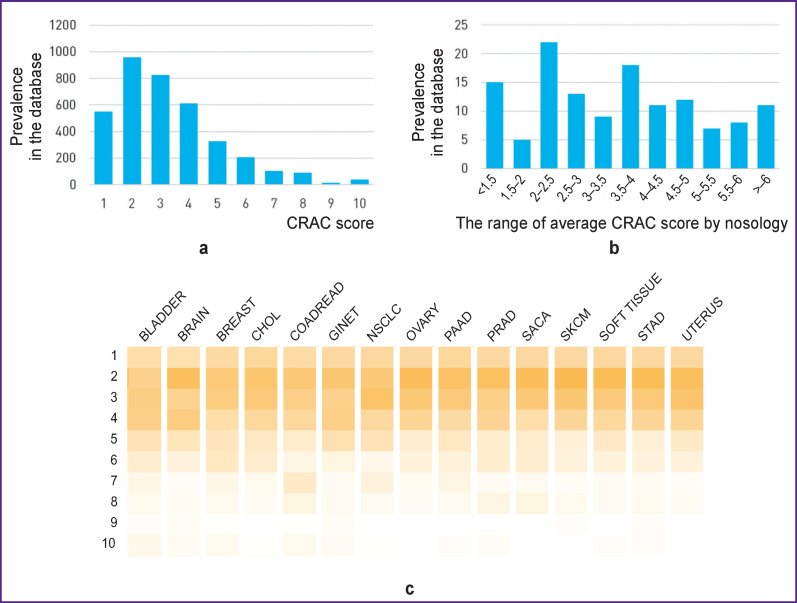
Description of the CRAC database: (a) distribution of CRAC scores characterizing 233 biomarkers in 15 nosologies; (b) distribution of the number of genes by scores average among CRAC nosologies; (c) distribution of CRAC scores by nosologies in the database neuroendocrine tumors of the gastrointestinal tract (n=2).

Biomarker–tumor type associations with CRAC scores in the range of 1–3 have the highest (n=2719 out of 3495; 77.8%) representation, with the highest CRAC scores of 9 and 10 have the lowest one (n=52 out of 3495; 1.5%) (Figure 2 (a)). The largest (n=40 out of 134; 29.8%) number of genes, in which genomic alterations are presented in the database, are in the middle range of 2–2.5; 3.5–4 by the nosology of CRAC scores (Figure 2 (b)).

### Retrospective analysis of reports on the results of comprehensive molecular profiling

We retrospectively analyzed the data obtained from the results of extended NGS-based molecular profiling in 208 patients with various nosologies, as well as, if available, reports with recommendations for therapy. The alterations presented in the report were reinterpreted, each abnormality was assigned a CRAC score. The reanalysis took into account the alterations that were not reported as potentially clinically significant.

The representation of nosologies among the analyzed sample of patients is as follows:

colorectal cancer (n=49);

adenocarcinoma of the pancreas (n=34);

breast cancer (n=23);

non-small cell lung cancer (n=21);

ovarian cancer (n=19);

adenocarcinoma of the stomach/esophagus (n=11);

soft tissue sarcoma (n=9);

cholangiocarcinoma (n=7);

salivary gland adenocarcinoma (n=6);

CNS tumors (n=6);

uterine body cancer (n=5);

bladder cancer (n=4);

prostate cancer (n=3);

skin melanoma (n=2);

A reanalysis of 210 clinically significant alterations, which are biomarkers for the potential effectiveness of molecularly matched therapy, was performed. Biomarkers of resistance were not taken into account. 79 biomarkers of evidence level I–III were detected according to the ESCAT system in 64 (30.7%) patients; 131 biomarkers of evidence level IV were identified in other 114 patients (55%) (Figure 3 (b)). The highest CRAC scores (8–10) corresponded to ESCAT evidence levels I and II. At the same time, no biomarker within the same level of evidence was represented by the same CRAC score; the largest range of CRAC scores was observed for biomarkers of evidence levels IIIA and IV — from 2 to 10 and from 1 to 9, respectively (Figure 3 (a)).

**Figure 3.**
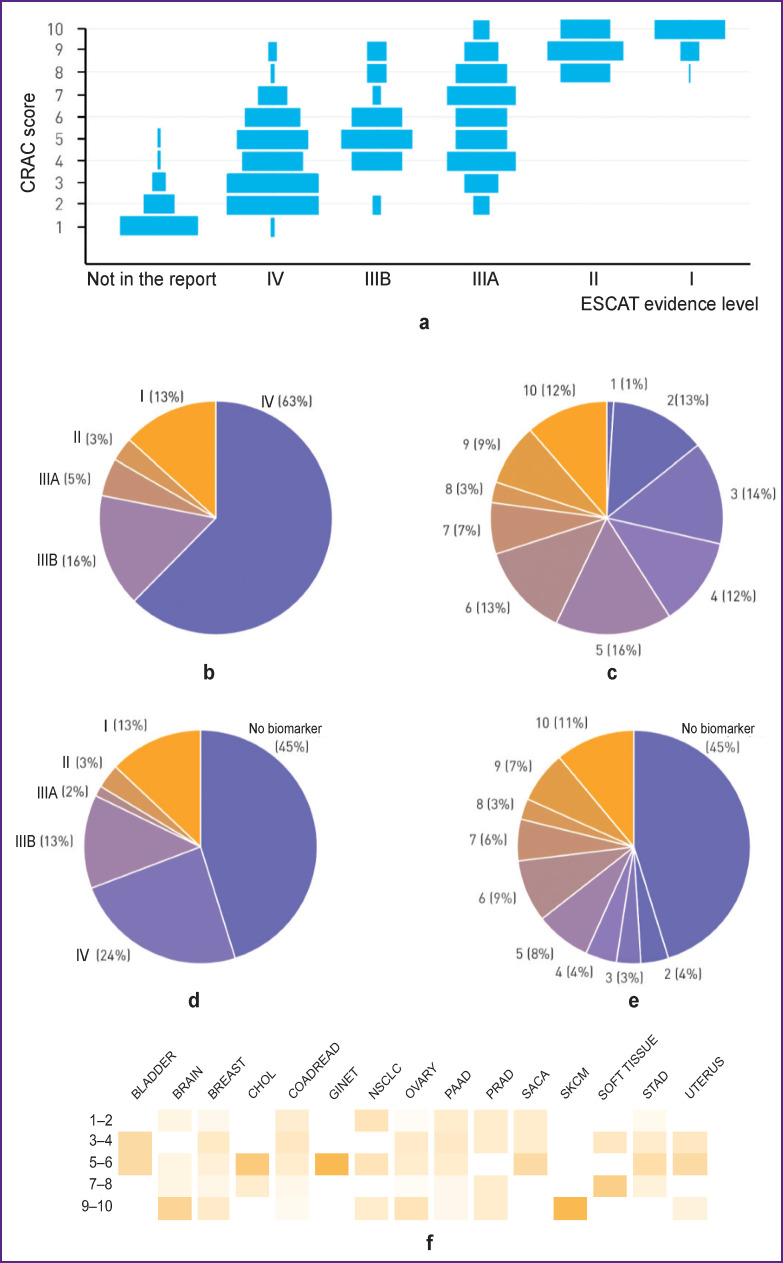
Use of the ESCAT levels of evidence and CRAC database to characterize biomarkers found in 208 patients with molecular profiling: (a) distribution of CRAC scores for different ESCAT levels of evidence that were reported on the results of molecular profiling, as well as those that were not put down in the report; (b) distribution of the ESCAT levels of evidence among biomarkers (n=210) found in the study population; (c) distribution of CRAC scores among all (n=305) biomarkers found in the study population; (d) occurrence of biomarkers among all patients and their corresponding ESCAT levels of evidence (if more than one biomarker was detected in one patient, only the one with the highest level of ESCAT evidence was taken into account); (e) occurrence of biomarkers among all patients and their corresponding CRAC scores (if more than one biomarker was detected in one patient, only the one with the highest CRAC score was taken into account); (f) distribution of CRAC scores of the detected biomarkers in the study population for various nosologies

The smallest number of biomarkers (n=3; 1%) had a CRAC score of 1, while biomarkers with CRAC scores of 2, 3, 4, 5, 6, 10 were evenly distributed for the detected alterations (Figure 3 (c)). Within the studied tumor types, different CRAC scores were observed. Thus, only alterations with the same CRAC score were characteristic for melanoma of the skin and neuroendocrine tumors of the gastrointestinal tract, while in the tumor samples of patients with colorectal cancer, pancreatic adenocarcinoma, and breast cancer, alterations of all CRAC scores were detected (from 1 to 10) (Figure 3 (f)).

More than 1 biomarker of evidence level I–IV were found in 63 out of 208 patients (30.3%), 14 patients (6.7%) had more than 1 biomarker of evidence level I–III. Two or more biomarkers of the same level of evidence were found in 8 patients (3.8%) — ESCAT I–III (of which I — in 1 patient, II — in 2 patients, III — in 5 patients). Thirty-four patients (16.3%) had at least two biomarkers with ESCAT evidence level IV. The occurrence of biomarkers and their corresponding levels of evidence according to ESCAT (if more than one biomarker was detected in one patient, only the one with the highest level of evidence according to ESCAT was taken into account) are shown in Figure 3 (d). The use of CRAC scores made it possible to additionally identify 95 alterations with CRAC scores of 1–5 (Figure 3 (a)).

As a result of applying the created database, the number of patients with two biomarkers which were assigned the same CRAC score was 9 (4.3%). At the same time, when recording the most promising biomarkers (CRAC score of 4 and higher), 4 patients (1.9%) with two biomarkers with the same CRAC score were identified. The number of patients with the same CRAC scores relative to the number of patients with biomarkers with the same ESCAT levels was significantly lower, indicating a high discriminatory power of the database. The occurrence of biomarkers in the study population and their corresponding CRAC scores (when more than one biomarker was detected in one patient, only the one with the highest CRAC score was taken into account) are shown in Figure 3 (e).

## Discussion

During molecular tumor profiling, several alterations associated with the potential effectiveness of a particular therapeutic approach can be detected. The problem of ranking recommended therapy can be solved in various ways, one of them is the introduction of the level of evidence system. Level of evidence systems are also designed to answer the question of how reliably the detected alteration is associated with the potential effectiveness or failure of treatment, and serve as a guideline for prescribing therapy by a physician [17]. To date, the OncoKB and ESCAT systems of evidence levels are most widely used [10, 11]. However, there are several problems that limit their applicability in oncological clinical practice. The first problem is that different level of evidence systems are not equivalent, which limits their interchangeability. Thus, the same alteration and its association with the same drug may have a different level of evidence depending on the chosen system. Evidence level systems are based on the reliability and extensiveness of the evidence base [18], but do not always take into account the negative results or the nature of the alteration. Thus, the level of evidence for a biomarker and drug may not always reflect the real effectiveness of a particular therapeutic approach.

Despite the fact that there are recommendations for the presentation of molecular genetic findings [19], they are standardized, and therefore there is significant heterogeneity in the presentation of the results of molecular genetic profiling depending on the laboratory. In addition, the detected alterations and recommended therapy are not always ranked in any way, including using levels of evidence, which can make it difficult for oncologists to work with the reports received. As a result, the oncologist has more questions than answers. If the evidence base for the association between a biomarker and a drug is not described in detail, the physician has to collect information on his/her own [20].

There are a large number of knowledge bases aggregating various predictive biomarkers and their associated therapies, some of them are widely used in clinical practice [10, 12]. However, the use of such databases is not always effective due to insufficient information and relevance of the data presented, different ranking systems for various therapeutic approaches, as well as the degree of awareness of oncologists about the types of genomic alterations [16]. Knowledge bases show excellent filling up with biomarkers used in routine clinical practice (ESCAT I, OncoKB R1), which cannot be said about biomarkers with a lower level of evidence.

The work of oncologists in an interdisciplinary team with geneticists, biologists, and bioinformaticians to form the most correct treatment strategy based on the results of molecular genetic studies is widespread in foreign oncological centers [21-23]. The work of molecular tumor boards has a direct impact on the outcomes of patient treatment, significantly improving them [22, 24].

In the context of the inaccessibility of advice by specialists in tumor biology, one of the solutions to the above problems can be a database on the clinical significance of the detected genomic alterations, which does not require deep knowledge in genetics and genomics. This is a tool that can help a physician to understand in a short period of time with minimal costs whether it is worth paying attention to the detected abnormality and, if so, what clinical effect will be from administering molecularly matched therapy to a patient with a detected biomarker. The database we have created answers the most common questions that arise before attending the physician: how likely molecularly matched therapy will be administered when a relevant alteration is detected; if several alterations are found, which one should be paid attention to first, i.e. how to rank the discovered findings with a high discriminatory power.

In our database, each biomarker is assigned a specific numerical score from 1 to 10, which may vary depending on the nosology and type of alteration. The score presented for biomarker and nosology is a numerical reflection of the answer to the question of what percentage of patients with a certain type of tumor and a certain biomarker can be candidates for appropriate therapy if this and only this biomarker is detected, and what will be the outcome of the administration of such therapy. For example, for variants of the *TP53* gene, regardless of nosology, number 1 (the minimum score) will appear in the database. Alterations in the *TP53* gene are among the most common ones in solid tumors [25, 26], however, a clear predictive role of such alterations in relation to available molecularly matched therapy has not been established [27, 28]. Thus, the prospects of this biomarker remain minimal today. The opposite, positive example is an alteration in exon 19 of the *EGFR* gene, in particular, for non-small cell lung cancer. Targeting such alterations with small molecule tyrosine kinase inhibitors of *EGFR* has long been in clinical practice and demonstrates a significant improvement in clinical parameters and patient survival [29-32]. In this regard, alterations in exon 19 of the *EGFR* gene in lung cancer are a promising biomarker, which reflects the maximum scores in the database.

Technically, the database can be used as follows: go to the website https://crac.oncoatlas.ru/, enter a gene of interest, and the system will generate CRAC scores for various types of alterations in this gene, depending on nosology.

## Conclusion

The CRAC database can be a useful tool in the hands of an oncologist. The developed knowledge base will facilitate answering the question of the prospects of the target based on the results of comprehensive molecular tumor profiling. In case of detection of several alterations, the use of CRAC scores will give an opportunity to select the most suitable target in terms of the prospects and knowledge of the biomarker. Finally, the CRAC database will help one to select the optimal amount of testing for the patient, depending on the tumor type, so as not to miss clinically significant alterations. Working with the database will save time when interpreting the results of molecular profiling by an oncologist, moreover, it does not require deep knowledge of molecular mechanisms of carcinogenesis.
